# Association between breastfeeding indicators and early childhood development: results from the *Brazilian National Survey on Child Nutrition*

**DOI:** 10.1590/0102-311XEN244225

**Published:** 2026-10-26

**Authors:** Nathalia Cristina Freitas-Costa, Davi Borges Damasceno, Juliana Vieira de Castro Mello, Raquel Machado Schincaglia, Dayana Rodrigues Farias, Nadya Helena Alves-Santos, Paula Normando, Inês Rugani Ribeiro de Castro, Gilberto Kac

**Affiliations:** 1 Instituto de Nutrição Josué de Castro, Universidade Federal do Rio de Janeiro, Rio de Janeiro, Brasil.; 2 Faculdade de Nutrição, Universidade Federal de Goiás, Goiânia, Brasil.; 3 Instituto de Ciências da Saúde, Universidade Federal do Pará, Belém, Brasil.; 4 Instituto de Nutrição, Universidade do Estado do Rio de Janeiro, Rio de Janeiro, Brasil.

**Keywords:** Breast Feeding, Infant Nutrition, Health Surveys, Aleitamento Materno, Nutrição Infantil, Inquéritos de Saúde, Lactancia Materna, Nutrición Infantil, Encuestas de Salud

## Abstract

We evaluated the association between breastfeeding indicators and early childhood development (ECD) among Brazilian children. Data from 5,766 children aged 1-23 months from the ENANI-2019 were analyzed. Breastfeeding indicators followed WHO recommendations: (1) 1-5 months: exclusive, predominant, or mixed breastfeeding, complementary feeding, and no breastfeeding; (2) 1-23 months: breastmilk intake and baby bottle use on the previous day. ECD was evaluated with the SWYC-BR. Developmental age was estimated using graded response models, and the developmental quotient (DQ) was calculated by dividing developmental age by chronological age. Multiple linear regression models were fitted. The mean (95%CI) DQ was higher among exclusively breastfed children aged 1-5 months [1.94 (1.72; 2.16)] than those receiving complementary feeding [1.56 (1.46; 1.67)]. Among children aged 1-23 months, DQ was higher in those receiving breastmilk [1.29 (1.24; 1.34)] than those not receiving it [0.99 (0.97; 1.00)], and in those who did not use a baby bottle on the previous day [1.38 (1.31; 1.45)] than in those who did [1.21 (1.17; 1.25)]. In the multiple regression models (β and 95%CI), exclusive breastfeeding was positively associated with DQ among children aged 1-5 months (β = 0.284; 0.093; 0.476, p = 0.004). Among children 1-23 months, receiving breastmilk (β = 0.195; 0.138; 0.252, p < 0.001) and not using a baby bottle on the previous day (β = 0.165; 0.093; 0.237, p < 0.001) were positively associated with DQ. Breastfeeding practices recommended by the WHO were positively associated with DQ.

## Resumo

Avaliou-se a associação entre indicadores de amamentação e desenvolvimento na primeira infância (DPI) em 5.766 crianças brasileiras de 1-23 meses do ENANI-2019. Os indicadores de amamentação seguiram as recomendações da OMS: (1) 1-5 meses: aleitamento materno exclusivo, predominante ou misto, alimentação complementar e ausência de aleitamento materno; (2) 1-23 meses: ingestão de leite materno e uso de mamadeira no dia anterior. O DPI foi avaliado pela SWYC-BR. A idade de desenvolvimento foi estimada por modelos de resposta graduada, e o quociente de desenvolvimento (QD) foi calculado dividindo-a pela idade cronológica. Foram ajustados modelos de regressão linear múltipla. O QD médio (IC95%) foi maior entre crianças de 1-5 meses em aleitamento materno exclusivo [1,94 (1,72; 2,16)] comparado àquelas em alimentação complementar [1,56 (1,46; 1,67)]; maior naquelas que recebiam leite materno [1,29 (1,24; 1,34)] *vs*. as que não recebiam [0,99 (0,97; 1,00)], e naquelas que não utilizaram mamadeira no dia anterior [1,38 (1,31; 1,45)] vs. as que utilizaram [1,21 (1,17; 1,25)], em lactentes de 1-23 meses. Nos modelos de regressão múltipla (β e IC95%), entre crianças de 1-5 meses, o aleitamento materno exclusivo (β = 0,284; 0,093; 0,476; p = 0,004) associou-se positivamente ao QD. Entre crianças de 1-23 meses, receber leite materno (β = 0,195; 0,138; 0,252; p < 0,001) e não usar mamadeira (β = 0,165; 0,093; 0,237; p < 0,001) no dia anterior associaram-se positivamente ao QD. As práticas de amamentação recomendadas pela OMS associaram-se positivamente ao QD.

## Resumen

Se evaluó la asociación entre indicadores de lactancia materna y desarrollo en la primera infancia (DPI) en 5.766 niños brasileños de 1-23 meses de la ENANI-2019. Los indicadores siguieron las recomendaciones de la OMS: (1) 1-5 meses: lactancia materna exclusiva, predominante o mixta, alimentación complementaria y sin lactancia; (2) 1-23 meses: consumo de leche materna y uso de biberón el día anterior. El DPI se evaluó con la SWYC-BR; el cociente de desarrollo (CD) se calculó dividiendo la edad de desarrollo, estimada mediante modelos de respuesta graduada, por la edad cronológica. El CD medio (IC95%) fue mayor en niños de 1-5 meses con lactancia materna exclusiva [1,94 (1,72; 2,16)] que con alimentación complementaria [1,56 (1,46; 1,67)]; en niños de 1-23 meses, en los que recibían leche materna [1,29 (1,24; 1,34)] que en los que no [0,99 (0,97; 1,00)], y en los que no usaron biberón el día anterior [1,38 (1,31; 1,45)] que en los que sí [1,21 (1,17; 1,25)]. En la regresión lineal múltiple (β e IC95%), la lactancia materna exclusiva se asoció positivamente con el CD en niños de 1-5 meses (β = 0,284; 0,093; 0,476, p = 0,004). En niños de 1-23 meses, recibir leche materna (β = 0,195; 0,138; 0,252, p < 0,001) y no usar biberón (β = 0,165; 0,093; 0,237, p < 0,001) el día anterior se asociaron positivamente con el CD. Las prácticas de lactancia recomendadas por la OMS se asociaron positivamente con el CD.

## Introduction

Exclusive breastfeeding for the first six months of life and continued breastfeeding until two years of age are recommended by the World Health Organization (WHO) and the United Nations Children’s Fund (UNICEF) ^1^. Breastfeeding has been investigated as one of the main factors determining early childhood development (ECD) ^2^ ^,^ ^3^. The WHO Nurturing Care Framework for ECD states that poverty, low maternal education, exposure to toxic stress, low birth weight, poor infant growth, absent or suboptimal breastfeeding, and inadequate nutrition are associated with suboptimal ECD ^4^.

Breast milk provides nutrients necessary for child growth and development ^5^. It also contains bioactive compounds, including polyunsaturated fatty acids, oligosaccharides, and immune factors, which have been associated with improved child development ^6^ ^,^ ^7^. Additionally, breastfeeding appears to be associated with socioemotional development and ECD stimuli ^8^. Mother-infant bonding and the parent-child attachment relationship established during breastfeeding are thought to be among the components associated with better overall and socioemotional ECD ^9^ ^,^ ^10^. Furthermore, continued breastfeeding is associated with responsive parenting practices, which can promote ECD ^11^ ^,^ ^12^.

Evidence consistently suggests a positive association between breastfeeding and cognitive development and intelligence ^8^ ^,^ ^13^ ^,^ ^14^. However, the relationship between breastfeeding and overall ECD, as well as other developmental domains (such as motor, language, emotional, and social), remains unclear ^8^ ^,^ ^13^ ^,^ ^15^. Moreover, findings from Brazil remain scarce and are limited to breastfeeding during the first 12 months of life and to regional data ^14^ ^,^ ^16^ ^,^ ^17^ ^,^ ^18^.

We previously demonstrated that Brazilian children face inequitable environments associated with lower levels of ECD ^19^, and that breastfeeding rates in Brazil remain below the targets established by the WHO and UNICEF for 2030 ^20^. In this context, and considering the UNICEF 2030 targets and the Sustainable Development Goals, expanding knowledge about the relationship between breastfeeding and ECD may not only contribute to the scientific evidence but also provide a foundation for informing public policies on this issue in Brazil and other low- and middle-income countries. Therefore, this study aims to evaluate the association between breastfeeding indicators and ECD in a representative sample of Brazilian children under five years of age.

## Methods

### Study design and participants

Data from the *Brazilian National Survey on Child Nutrition* (ENANI-2019) were used. The ENANI-2019 study population consisted of households with at least one child under five years of age, excluding: (i) Indigenous communities living in villages; (ii) foreign children living in households where Portuguese was not spoken; (iii) children with any condition that prevented anthropometric measurements; and (iv) children living in hotels, boarding houses, orphanages, and hospitals ^21^.

Data collection for ENANI-2019 occurred between February 2019 and March 2020 in 123 municipalities across the 26 states and the Federal District of Brazil ^22^. The study used a complex, stratified, clustered sampling design and applied sampling weights that were calculated and calibrated to account for unequal selection probabilities and non-response. This calibration procedure aimed to minimize potential biases arising from differential non-response and to preserve the representativeness of the study population ^22^.

Data from 14,558 children under five years of age residing in 12,524 households were included. Children aged < 1 month (n = 249) were ineligible for the ECD assessment. Those aged ≥ 24 months (n = 8,236) and cases with missing data on maternal age (n = 4) or parity (n = 303) were excluded from the analysis. Thus, data from 5,766 children aged 1-23 months were included, comprising 1,683 children aged 1-5 months and 4,083 children aged 6-23 months (Figure 1).

**Figure 1:**
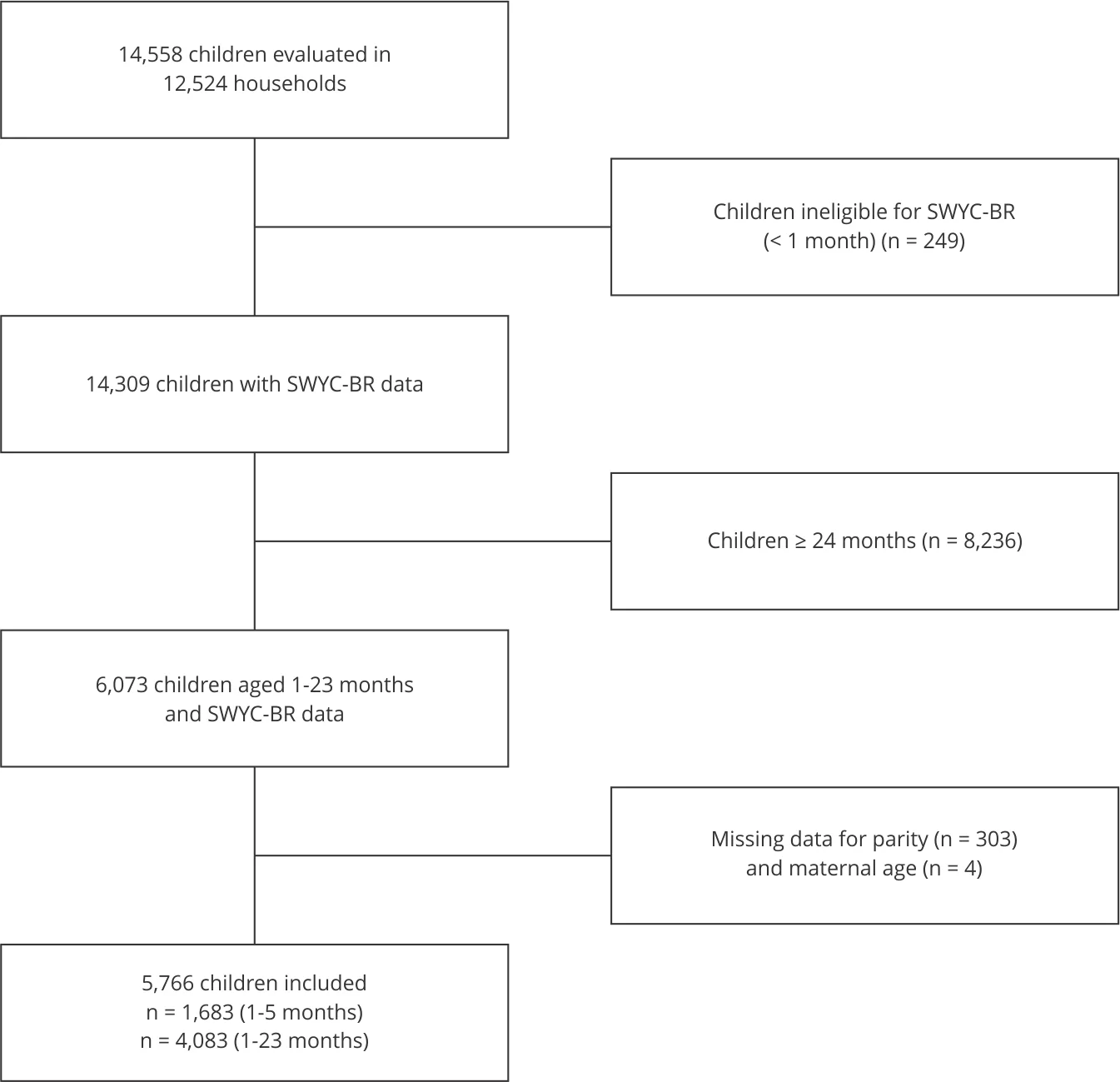
Flow diagram of children aged 1-23 months evaluated in *Brazilian National Survey on Child Nutrition* (ENANI-2019).

SWYC-BR: Brazilian version of the *Survey of Well-being of Young Children* milestones questionnaire.

### Early childhood development assessment

ECD was assessed using the Brazilian version of the *Survey of Well-being of Young Children* (SWYC-BR) milestones questionnaire. This tool was developed in 2011 and validated for the United States population in 2013 ^23^. The SWYC was translated, adapted, and validated for Brazilian children in 2019 ^24^ ^,^ ^25^. We assessed the internal consistency of the SWYC-BR using ENANI-2019 data, which showed adequate performance ^26^.

The SWYC-BR comprises 54 developmental milestones with 12 sets of 10 specific items per age group. These age groups are 1-3, 4-5, 6-8, 9-11, 12-14, 15-17, 18-22, 23-28, 29-34, 35-46, 47-58, and 59-65 months. The milestones items are easy to read and administer and do not require specialized professionals or specific materials or equipment. The questions refer to daily activities for each age group and assess progress in one or more dimensions of ECD. Three response options were provided: “not at all”, “somewhat”, or “very much”.

Based on the child’s date of birth reported during the interview, the ENANI-2019 data collection system automatically selected the developmental milestones appropriate for their age group. For preterm children (< 37 weeks of gestation) under two years of age, the questionnaire corresponding to their corrected age was used. This was determined by subtracting the child’s gestational age at birth from 40 weeks (full-term) and then subtracting that difference from the child’s chronological age at the time of assessment. Additional information on the corrected age in the ECD assessment has been described elsewhere ^26^.

### Developmental quotient

The developmental quotient (DQ) was used as an indicator of the achievement of developmental milestones expected for an age group. It was calculated by dividing developmental age by chronological age. Developmental age was estimated using item response theory and graded response models in Mplus, version 7 (https://www.statmodel.com/), with the full-information method and a complex sampling design ^27^. The item response theory model enables the evaluation of each item in an instrument, rather than only the final score. It reduces bias by accounting for differences in item difficulty, making it easier to reach the maximum score within each age group. This methodology is described in more detail in Freitas-Costa et al. ^26^ and has previously been used to assess ECD using the SWYC ^23^ ^,^ ^26^ ^,^ ^28^ and the Denver Test ^29^.

The DQ reflects the achievement of developmental milestones expected for age; a DQ of 1 indicates that these milestones were attained. Values < 1 and > 1 suggest that milestone attainment was below and above expectations for age, respectively ^23^.

### Breastfeeding indicators

Breastfeeding indicators were calculated from responses to a structured 40-item questionnaire about the child’s feeding practices on the day before the interview. This instrument was designed to evaluate indicators of feeding practices for children under two years of age, as proposed by the WHO and the Brazilian Ministry of Health. The set of indicators that can be calculated from this questionnaire includes the timely introduction of foods, dietary diversity, provision of vitamin A and iron sources, and consumption of ultra-processed foods ^30^. The mother/caregiver was asked about the child’s breastmilk, food, and liquid consumption, e.g., “Did [child name] drink breastmilk yesterday?”. The response options were “yes”, “no”, and “does not know/did not want to answer”. More details about this questionnaire can be found in Lacerda et al. ^30^.

To assess breastfeeding practices, we used age-specific indicators. For children aged 1-5 months, the WHO breastfeeding indicator comprised the following five categories: (a) exclusive breastfeeding - received only breastmilk, without tea, juice, or water; (b) predominant breastfeeding - received breastmilk, water, teas, and/or juice; (c) mixed breastfeeding - received breastmilk and infant formula, regardless of the consumption of water, juice, and/or tea; (d) complementary feeding - received breastmilk and any solid or semi-solid food; (e) no-breastfeeding - did not receive breastmilk. It is important to note that the predominant breastfeeding indicator is no longer recommended for routine reporting ^31^. However, we aimed to create a mutually exclusive categorical variable that describes infant feeding practices. The predominant breastfeeding category was included because it enables the accurate classification of children who are breastfed but also consume water, tea, or juice, without consuming infant formula or solid foods, and therefore do not fit into any other category. Additionally, exclusive breastfeeding was analyzed as a dichotomous variable, with exclusive breastfeeding corresponding to the exclusive breastfeeding category in the WHO indicator and non- exclusive breastfeeding encompassing all other WHO breastfeeding categories ^31^.

For children aged 1-23 months, the indicators breastmilk intake (yes, no) and baby bottle use (yes, no) were used. Breastmilk intake indicates whether the child received breastmilk on the day before the interview, regardless of the consumption of other foods or beverages. Baby bottle use refers to whether the child was fed using a bottle on the day before the interview ^31^.

### Characteristics of the household, caregiver, and child

The following socioeconomic characteristics were considered: infant sex and age (1-5, 6-11, or 12-23 months), mother/caregiver educational level (0-7, 8-10, or ≥ 11 years), caregiver marital status (lives with partner: yes, no), maternal/caregiver occupation (employed, unemployed, housewife, student, other), maternal age (14-20, 20-30, > 30 years), participation in the Brazilian Income Transfer Program (yes, receives another benefit, does not receive any benefit), and monthly per capita family income (< 62.2, 62.2-124.4, 124.5-248.7, > 248.7 USD). Per capita family income was calculated by dividing total monthly family income by the number of people living in the household. Then, the value was converted to Brazilian minimum wages during the study period (BRL 998.00 in 2019 and BRL 1,045.00 in 2020), and converted using the United States dollar exchange rate (BRL 4.013 = USD 1) for December 2019.

### Birth and health variables

Type of delivery (vaginal, planned/elective caesarean, emergency caesarean), parity (primiparous, multiparous), usual access to health services (public primary care, other public services, private), breastfeeding in the first hour after birth (yes, no), preterm birth (< 37 gestational weeks: yes, no), and birth weight for gestational age were evaluated using a structured questionnaire. Birth weight percentiles according to gestational age and sex were calculated using the International Fetal and Newborn Growth Consortium for the 21st Century (Intergrowth-21st) recommendations ^32^. These percentiles were used to classify newborns as small for gestational age (SGA, percentile < 10), adequate for gestational age (AGA, percentile 10-90), or large for gestational age (LGA, percentile > 90).

### Statistical analysis

The distribution of variables was evaluated using histograms and scatter plots. Mean DQ, frequency, and 95% confidence intervals (95%CI) were used to describe the data. Linear regression analysis was performed to evaluate the association between chronological age and DQ and to assess the independent interactions between chronological age in months and breastfeeding indicators. Multiple linear regression models were constructed to investigate the association between breastfeeding and DQ by estimating the beta coefficient, its respective 95%CI, and the p-value. In a previous study, we showed that the DQ of Brazilian children declines with chronological age ^26^. Therefore, we also assessed how chronological age interacts with breastfeeding indicators in relation to DQ using linear regression models.

A directed acyclic graph (DAG) was used to identify the minimum sufficient set of variables to adjust for the association between breastfeeding and ECD (Figure 2) ^33^. The suggested adjustment variables were maternal/caregiver education (years), parity, maternal/caregiver occupation, birth weight by gestational age, type of delivery, bottle-feeding, access to health services, and breastfeeding in the first hour.

**Figure 2:**
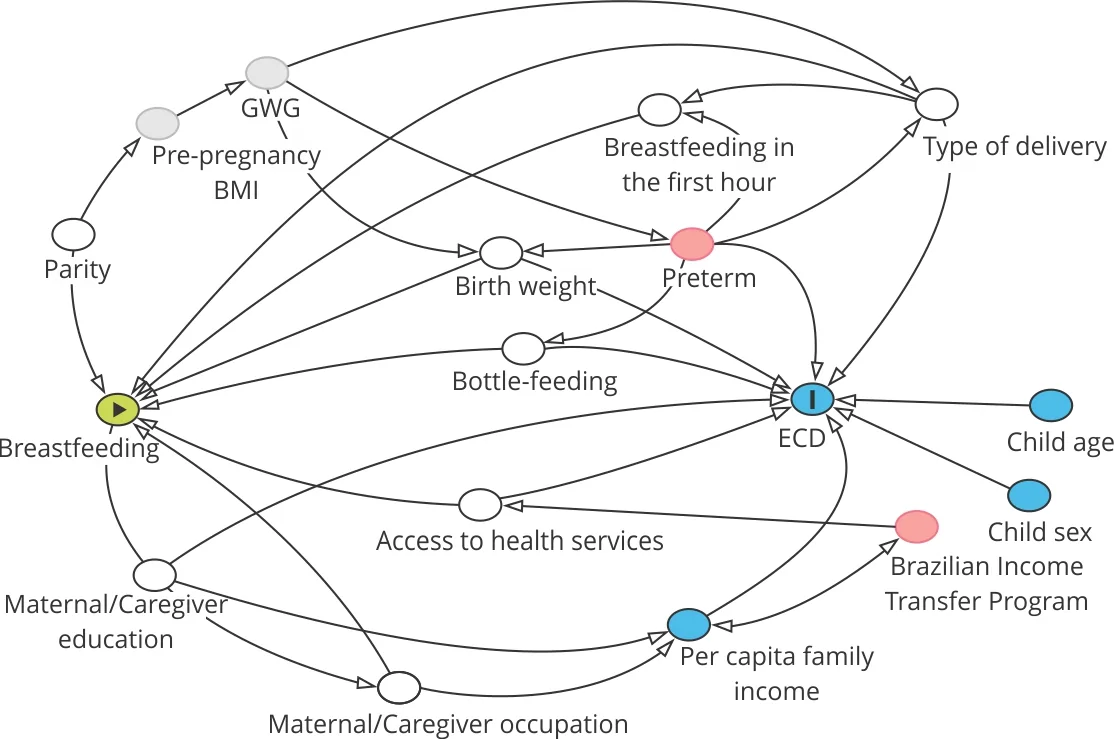
Directed acyclic graph on breastfeeding practice and early childhood development (ECD).

BMI: body mass index; GWG: gestational weight gain. Note: minimum adjustments suggested by the directed acyclic graph: maternal/caregiver education (years), parity, maternal/caregiver occupation, birth weight by gestational age, type of delivery, bottle-feeding, access to health services, and breastfeeding in the first hour. Blue circles: ancestor of the outcome; pink circles: ancestor of exposure and outcome; grey circle: unobserved variable; white circle: adjustment variables.

The statistical analyses were conducted in the Jupyter collaborative environment and in R (http://www.r-project.org) using the *srvyr* and *survey* packages. Calibrated sample weights were used to account for the complex sampling design, which incorporates stratification, clustering, and unequal selection probabilities. Differences in mean DQ and frequencies across the selected variables were considered statistically significant when the 95%CIs did not overlap. Given the large sample size, which may increase the likelihood of statistically significant results in traditional hypothesis tests, confidence intervals provide a more informative assessment of the precision of the differences ^34^. For all other analyses, statistical significance was considered when p < 0.05.

### Ethical approval

ENANI-2019 was approved by the research ethics committee of the Clementino Fraga Filho University Hospital, Federal University of Rio de Janeiro (number: 89798718.7.0000.5257). Participation in the research occurred upon written informed consent by the child’s parent or caregiver, and after hearing an explanation about the study’s ethical issues.

## Results

The mean (95%CI) DQ for children aged 1-23 months was 1.29 (1.25; 1.34). Higher mean DQ was observed among children aged 1-5 [1.78 (1.66; 1.90)] and 6-11 months [1.28 (1.23; 1.32)] than among those aged 12-23 months [1.07 (1.03; 1.10)], and among children whose mothers/caregivers had a higher level of education [≥ 11 years: 1.34 (1.28; 1.39)] than those whose mothers/caregivers had 0-7 years of schooling [1.21 (1.15; 1.26)] (Table 1). The prevalence of exclusive breastfeeding, predominant breastfeeding, mixed breastfeeding, and complementary feeding among children aged 1-5 months was 42.6%, 14.3%, 9.1%, and 20.7%, respectively (Figure 3a). Among children aged 1-23 months, 61.5% (95%CI: 58.5; 64.5) received breastmilk the day before the interview, and the prevalence of baby bottle use was 51.6% (47.9; 55.2) (Figures 3c and 3d).

**Table 1:** Mean developmental quotient according to select variables for children aged 1-23 months evaluated in *Brazilian National Survey on Child Nutrition* (ENANI-2019) (n = 5,766).

Variable	Sample distribution (%)	Mean	95%CI
Brazil	100.0	1.29	1.25; 1.34
Sex			
Male	51.4	1.26	1.20; 1.32
Female	48.6	1.33	1.28; 1.38
Age group (months)			
1-5	23.7	1.78^ab^	1.66; 1.90
6-11	26.0	1.28^ac^	1.23; 1.32
12-23	50.3	1.07^bc^	1.04; 1.11
Maternal age (years)			
14-20	10.6	1.29	1.23; 1.36
20-30	56.8	1.33	1.26; 1.39
> 30	32.6	1.24	1.19; 1.29
Maternal/Caregiver education (years)			
0-7	20.5	1.21^a^	1.15; 1.26
8-10	22.6	1.27	1.18; 1.35
≥ 11	56.9	1.34^a^	1.28; 1.39
Marital status (lives with partner)			
Yes	75.2	1.27	1.22; 1.32
No	24.8	1.37	1.29; 1.44
Maternal/Caregiver occupation			
Employed	36.0	1.29	1.24; 1.34
Unemployed	25.5	1.30	1.25; 1.35
Housewife	35.8	1.28	1.21; 1.34
Student or other	2.7	1.47	1.10; 1.84
Per capita family income (USD) *			
< 62.2	28.6	1.30	1.22; 1.38
62.2-124.4	33.2	1.24	1.19; 1.30
124.5-248.7	24.7	1.35	1.28; 1.41
> 248.7	13.5	1.30	1.20; 1.40
Brazilian Income Transfer Program			
Yes	35.6	1.23	1.18; 1.27
Receives other benefit	4.9	1.29	1.17; 1.42
Does not receive any benefit	59.5	1.33	1.26; 1.40
Parity			
Primiparous	40.4	1.32	1.26; 1.39
Multiparous	59.6	1.27	1.22; 1.32
Preterm birth (< 37 gestational weeks)			
Yes	9.8	1.37	1.29; 1.44
No	90.2	1.29	1.24; 1.33
Birth weight by gestational age (Intergrowth 21^st^) ^32^			
Small for gestational age (< percentile 10)	10.0	1.26	1.14; 1.39
Appropriate for gestational age (percentile 10-percentile 90)	74.3	1.30	1.26; 1.35
Large for gestational age (> percentile 90)	15.4	1.26	1.19; 1.33
Type of delivery			
Vaginal	53.6	1.31	1.25; 1.36
Planned/Elective caesarean	19.2	1.34	1.27; 1.41
Emergency caesarean	27.2	1.24	1.18; 1.29
Breastfeeding in the first hour			
Yes	61.9	1.31	1.25; 1.37
No	38.1	1.27	1.23; 1.31
Access to health services **			
Public (primary care unit)	74.4	1.29	1.23; 1.34
Public (others)	5.6	1.27	1.12; 1.41
Private	19.1	1.33	1.25; 1.41

95%CI: 95% confidence interval.

Note: “abc” upper letters indicates mean differences across variable categories, based on the lack of 95%CI overlap; that is, categories sharing the same letter show differences between categories.

* Family monthly income was measured according to the minimum wage during the period in which the research took place, in 2019, BRL 998.00, and in 2020, BRL 1045.00, and converted according to the dollar exchange rate (BRL 4.013 = USD 1) in 2019;

** A category of other represents < 1% of the population and was not shown in the results.

**Figure 3:**
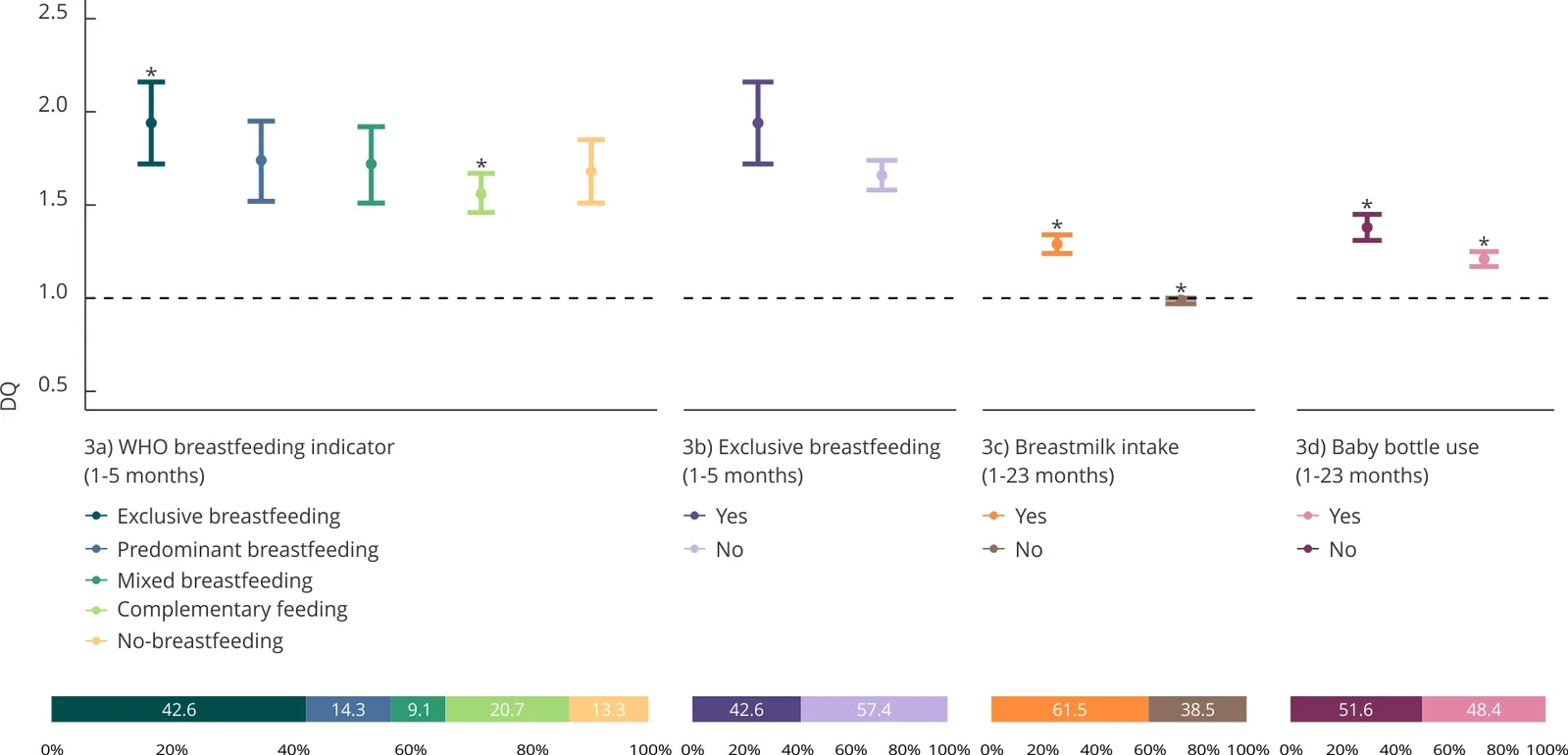
Mean developmental quotient (DQ) according to breastfeeding indicators for children aged 1-5 and 1-23 months evaluated in *Brazilian National Survey on Child Nutrition* (ENANI-2019) (n = 5,766).

WHO: World Health Organization. Note: the DQ was determined using the milestones questionnaire from the Brazilian adaptation of the *Survey of Well-being of Young Children* (SWYC-BR). Estimating developmental age considers the child’s chronological age and the achieved developmental milestones. The DQ is computed by dividing the developmental age by the chronological age. A DQ = 1 means the attainment of expected age milestones, while DQ < 1 and > 1 indicate that specific age milestones were reached below or above expectations, respectively. 3a) The WHO breastfeeding indicator was defined with the following five categories: (i) exclusive breastfeeding - received only breastmilk, without tea, juice, or water; (ii) predominant breastfeeding - received breastmilk, water, tea, and/or juice; (iii) mixed breastfeeding - received breastmilk and infant formula, regardless of the consumption of water, juice, and/or tea; (iv) complementary feeding - received breastmilk and any solid or semi-solid food; (v) No-breastfeeding - does not receive breastmilk. 3b) Exclusive breastfeeding was defined for children who received only breastmilk, and those who received any other food/liquid on the day before the interview were classified as non-exclusive breastfeeding. 3c) The breastmilk intake indicator refers to whether the child received breastmilk or not, regardless of other foods/drinks on the day before the interview. 3d) Baby bottle use refers to baby bottle use on the day before the interview. Color bars for the four breastfeeding indicators represent the frequency distribution for the indicator-specific categories. * Statistically significant differences in the mean DQ across breastfeeding practice categories, based on the absence of 95% confidence interval overlap.

The mean DQ among children aged 1-5 months who were exclusively breastfed was 1.94 (1.72; 2.16) and was significantly higher than that of children receiving complementary feeding [1.56 (1.46; 1.67)] (Figure 3a). Conversely, we found no difference in mean DQ according to the exclusive breastfeeding indicator [exclusive breastfeeding: 1.94 (1.72; 2.16), non-exclusive breastfeeding: 1.66 (1.58; 1.74), Figure 3b]. The mean DQ among children aged 1-23 months differed between those who received breastmilk the day before the interview [1.29 (1.24; 1.34)] and those who did not [0.99 (0.97; 1.00)] (Figure 3c). Mean QD was significantly higher among infants who did not use a baby bottle the day before the interview [1.38 (1.31; 1.45)] than among those who did [1.21 (1.17; 1.25)] (Figure 3d).

Exclusive breastfeeding was positively associated with DQ for children aged 1-5 months in the fully adjusted model compared to non-exclusive breastfeeding (β = 0.284; 95%CI: 0.093; 0.476; p = 0.004). Among children aged 1-23 months, receiving breastmilk the day before the interview (β = 0.195; 95%CI: 0.138; 0.252; p < 0.001) and not using a baby bottle (β = 0.165; 95%CI: 0.093; 0.237; p < 0.001) were also positively associated with DQ (Figure 4). In contrast, no statistically significant associations were observed between the WHO breastfeeding indicator categories and DQ (Supplementary Material; https://cadernos.ensp.fiocruz.br/ojs/index.php/csp/article/view/11922/21117).

**Figure 4:**
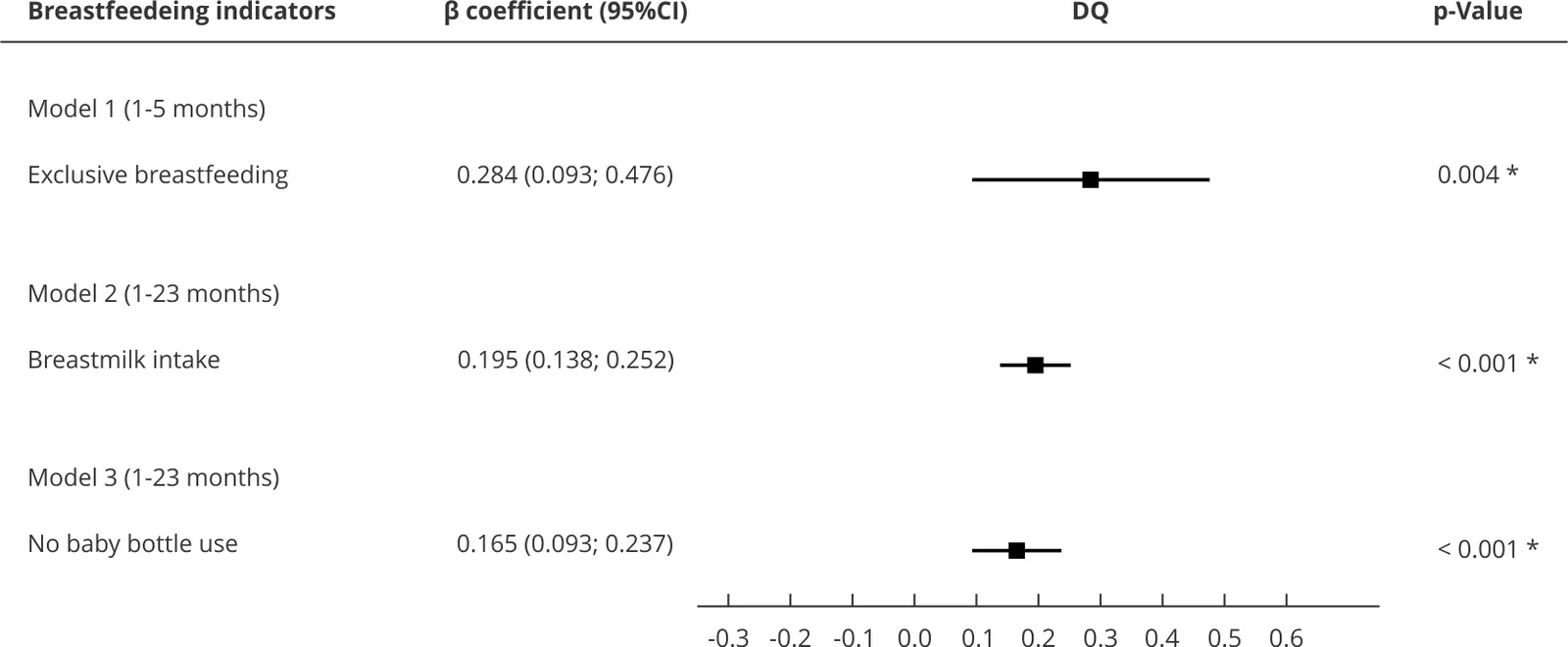
Association between breastfeeding indicators and developmental quotient (DQ) for children 1-5 and 1-23 months evaluated in *Brazilian National Survey on Child Nutrition* (ENANI-2019) (n = 5,766).

95%CI: 95% confidence interval. Note: model 1 - for infants aged 1-5 months (n = 1,683), using the indicator of exclusive breastfeeding, and “no exclusive breastfeeding” as a reference category; model 2 - for children aged 1-23 months (n = 5,766), using breastmilk intake the day before the interview indicator (yes, no), and “no” as a reference category; model 3 - for children aged 1-23 months (n = 5,766), using the indicator of baby bottle use the day before the interview (yes, no), and “yes” as a reference category. Models 1 and 2 were adjusted according to directed acyclic graph suggestions for maternal/caregiver education (years), parity, maternal/caregiver occupation, birth weight by gestational age, type of delivery, bottle-feeding, access to health services, and breastfeeding in the first hour. Model 3 was adjusted according to directed acyclic graph for the same variables of models 1 and 2, except for bottle-feeding. * Statistical significance based on the p-value < 0.05.

DQ decreased more markedly with increasing chronological age among children who received breastmilk the day before the interview (β: -0.022; 95%CI: -0.034; -0.011; p = 0.001) and those who did not use a baby bottle (β: -0.013; 95%CI: -0.024; -0.001; p = 0.027), compared to their counterparts (Supplementary Material; https://cadernos.ensp.fiocruz.br/ojs/index.php/csp/article/view/11922/21117).

## Discussion

This is the first study to evaluate the association between breastfeeding indicators and ECD in a representative sample of Brazilian children. The mean DQ for children aged 1-5 months who were exclusively breastfed was significantly higher than that of those receiving complementary feeding. We also observed significantly lower mean DQ values among children aged 1-23 months who had not received breastmilk or used a baby bottle the previous day, compared to those who had not. Moreover, in the adjusted multiple regression model, exclusive breastfeeding, receiving breastmilk, and not using a baby bottle the day before were positively associated with higher DQ. These results indicate that being exclusively breastfed for up to six months was associated with a 0.28-point higher DQ score than non-exclusively breastfed, while receiving breastmilk and not using a baby bottle were associated with 0.19- and 0.16-point higher DQ scores, respectively.

The relationship between breastfeeding and infant motor development scores has been studied, and the findings are consistent, showing higher scores among children with better breastfeeding practices ^13^ ^,^ ^14^ ^,^ ^35^ ^,^ ^36^. A meta-analysis of 14 studies published between 2001 and 2020 showed a positive association between exclusive breastfeeding and motor development ^13^. For the other domains of ECD, findings on the type of breastfeeding practice before six months remain inconsistent ^2^ ^,^ ^8^. The results from ENANI-2019 indicated that exclusive breastfeeding was positively associated with DQ, and several mechanisms may explain this association. Mothers who breastfeed tend to dedicate more time to nurturing care than mothers who use formula ^37^. The longer duration of maternal care is another mechanism that may explain this relationship, as an infant’s brain development is shaped by environmental interactions, especially those between mother and child ^38^. Consequently, breastfeeding may support brain development by its nutritional benefits, mother -baby bonding, and responsive parenting ^37^ ^,^ ^38^.

Little is known about the relationship between early food introduction before six months and ECD ^39^. In our study, children aged 1-5 months receiving complementary feeding had lower mean DQ values than those who were exclusively breastfed. The WHO guideline for complementary feeding recently highlighted that introducing complementary foods before six months is associated with long-term adverse outcomes, especially increased body mass index ^40^. Early introduction of complementary foods is associated with the cessation or reduction of exclusive breastfeeding and breastfeeding, often leading to the use of infant formula. Infants who are not breastfed tend to be fed on a fixed schedule, regardless of hunger and satiety cues, which may result in higher caloric intake and an increased risk of obesity ^41^. Excess weight may limit the attainment of motor milestones and is associated with alterations in metabolic and inflammatory pathways, which together may contribute to adverse ECD outcomes.

The ENANI-2019 results revealed that receiving breast milk and not using a baby bottle were positively associated with higher DQ scores among children aged 1-23 months. These results align with the scientific literature, which indicates that any breastfeeding type and longer breastfeeding duration are associated with lower ECD, either by increasing developmental scores or by reducing the risk of delay ^42^ ^,^ ^43^ ^,^ ^44^ ^,^ ^45^ ^,^ ^46^. Sánchez-Vincitore et al. ^3^ evaluated 699 Dominican children aged 12-36 months and found that having been breastfed was associated with higher overall ECD scores and higher language and fine motor development scores than among those who had not been breastfed. McCrory et al. ^44^ assessed 11,134 Irish children aged nine months and found a positive association between any type of breastfeeding and gross motor skills, fine motor skills, problem-solving, and personal and social development. Villar et al. ^46^ used data from a multicenter study evaluating 1,306 mother-child pairs with adequate health and nutritional conditions and found that longer exposure to breastmilk (≥ 5 months) led to higher scores for gross motor development, visual acuity, age at walking and running, and climbing stairs without assistance compared to those breastfed for < 5 months or those who had never received breastmilk.

The use of baby bottles, pacifiers, and other artificial nipples has also been described as a possible factor contributing to breastfeeding interruption ^18^ ^,^ ^47^ ^,^ ^48^. This may explain how using these devices worsens ECD. Furthermore, baby bottle use may be associated with the use of human milk substitutes, as well as with flour and other complementary foods commonly offered in Brazil. Pacifier and bottle use have also been investigated as possible contributors to speech, language, and cognitive development problems ^49^. Among children included in ENANI-2019, we observed that not using a baby bottle was positively associated with DQ. A possible hypothesis is that using pacifiers and bottles to comfort children reduces interaction between the child and the caregiver ^50^. Furthermore, speech delays associated with pacifier and bottle use may result from myofunctional alterations caused by the presence of the pacifier or bottle nipple in the oral cavity ^51^.

The current findings should be interpreted in light of some potential limitations. ENANI-2019 is a cross-sectional study; therefore, the interpretation of the observed relationships and their causal nature must be made with caution. However, when interpreted within established causal inference frameworks, such as *Hill’s Criteria for Causation* ^52^, the observed associations are supported by consistency across studies, biological plausibility, and coherence with the existing literature. Additionally, breastfeeding practices were assessed based on food consumption the day before the interview, which may not fully represent typical feeding patterns. Measures of variables related to child stimulation (such as responsive caregiving, play, and verbal interaction) were not available. However, these factors are directly related only to the outcome and are not part of the causal pathway under investigation; thus, they would not have been included in the multiple regression models. Future studies, including ENANI-2024, that incorporate indicators of child stimulation will enable a more comprehensive assessment of these dimensions.

Despite the highlighted limitations, the current results represent an innovative study and the first to assess ECD in a representative sample of the Brazilian population. ECD was assessed using the SWYC-BR milestones questionnaire, which was previously translated, adapted, and validated for use in Brazilian children ^25^. The instrument showed adequate internal consistency, and confirmatory factor analysis supported its unidimensional structure, consistent with that of the original tool ^23^ ^,^ ^24^ ^,^ ^26^. Furthermore, we used the DQ to assess ECD as a continuous measure that incorporates each developmental milestone and the chronological age at which it was achieved, rather than as a summed score and cutoff points, as used in most child development scales and studies ^28^. This approach made it possible to capture greater variability in developmental outcomes. These findings reinforce the importance of promoting adequate nutrition to ensure optimal ECD, as well as the need to better integrate public policies on nutrition and child development, especially for children aged 1-23 months, as highlighted by the WHO Nurturing Care Framework for ECD ^4^.

Furthermore, Brazil was a pioneer in establishing principles and guidelines for the formulation of public policy in early childhood by specific legislation ^53^ ^,^ ^54^. Thus, this study may contribute as a guide for early childhood policymakers. We emphasize that, in addition to promoting breastfeeding, public policies must support sustained breastfeeding. This includes: (1) increasing the number of baby-friendly hospitals and human milk banks; (2) providing paid maternity leave of at least six months, enabling exclusive breastfeeding for the first six months, as recommended by the WHO; (3) increasing the number of breastfeeding rooms in workplaces; (4) ensuring access to daycare/school facilities that offer breastmilk rather than promoting weaning, thereby supporting continued breastfeeding beyond six months; and (5) regulating and monitoring the marketing of human milk substitutes, which often contributes to early weaning.

In conclusion, adherence to breastfeeding practices recommended by the WHO was positively associated with ECD among Brazilian children, both in the restricted age range of 1-5 months and in the broader range of 1-23 months. These results reinforce the need to improve breastfeeding practices as short- and long-term interventions to promote ECD.

## Data Availability

The databases used in the study, including extraction codes, analyses, and results, are available in the repository: https://enani.nutricao.ufrj.br/materialinstrutivo-e-banco-de-dados/.
