# Supplementary material for: Association between breastfeeding indicators and early childhood development: results from the Brazilian National Survey on Child Nutrition
Source: Cad Saude Publica. 2026 Oct 26;42:e00244225. doi: 10.1590/0102-311XEN244225 (PMC13706636; doi:10.1590/0102-311XEN244225)
Supplement: Supplementary file 1 [file 1678-4464-csp-42-EN244225-s.pdf]

Supplementary Table 1 Association between the World Health Organization breastfeeding indicator categories and developmental quotient for children 1-5 months evaluated in ENANI-2019 (n = 1,683).

WHO BF categories	Coefficient	CI 95%		*p*-value
Non-BF		Reference		
Exclusive BF	0.258	-0.013	0.530	0.061
Predominant BF	0.039	-0.261	0.338	0.799
Mixed BF	-0.018	-0.307	0.271	0.902
Complementary feeding	-0.111	-0.321	0.099	0.300

Note: multiple linear regression models were performed for infants aged 1-5 months, using the World Health Organization (WHO) breastfeeding (BF) indicator, with no-BF as the reference category. The model was adjusted according to the DAG suggestions for maternal/caregiver education (years), parity, maternal/caregiver occupation, birth weight by gestational age, type of delivery, bottle-feeding, access to health services, and breastfeeding in the 1st hour. The Developmental Quotient (DQ) was determined utilizing the milestones questionnaire from the Brazilian adaptation of the Survey of Well-being of Young Children (SWYC-BR). Estimating developmental age considers the child's chronological age and the achieved developmental milestones. The Development Quotient (DQ) is computed by dividing the developmental age by the chronological age. A DQ=1 signifies the attainment of expected age milestones, while DQ < 1 and > 1 indicate that specific age milestones were reached below or above expectations, respectively. The World Health Organization (WHO) BF indicator was defined with the following five categories: a) Exclusive BF – received only breastmilk, without tea, juice, or water; b) Predominant BF – received breastmilk, water, teas and/or juice; c) Mixed BF – received breastmilk and infant formula, regardless of the consumption of water, juice and/or tea; d) Complementary feeding – received breastmilk and any solid or semi-solid food; e) No-BF- does not receive breastmilk.

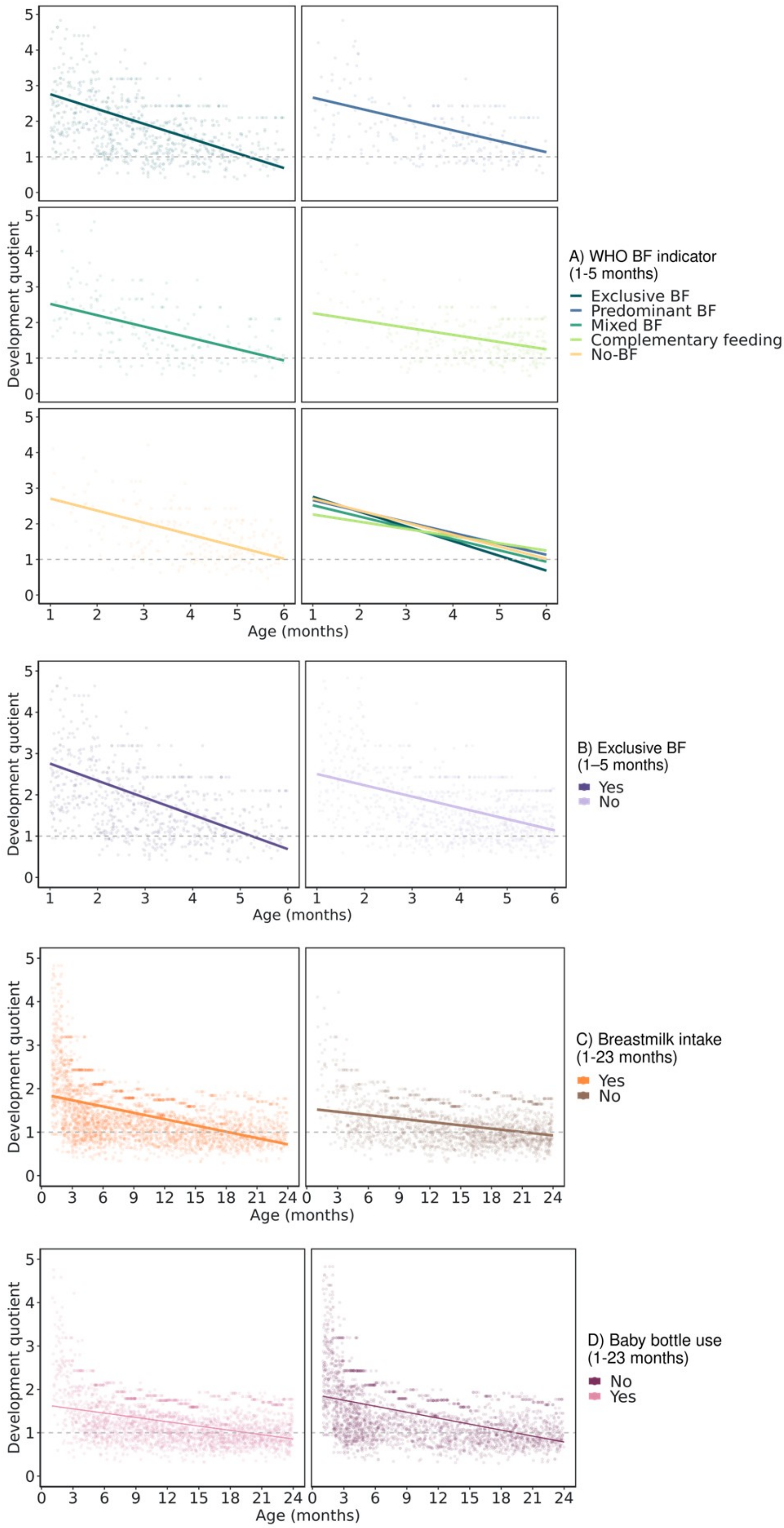

A) WHO BF indicator
(1-5 months)
Exclusive BF
Predominant BF
Mixed BF
Complementary feeding
No-BF
B) Exclusive BF
(1–5 months)
Yes
No
C) Breastmilk intake
(1-23 months)
Yes
No
D) Baby bottle use
(1-23 months)
No
Yes
Development quotient
Age (months)

Supplementary Figure 1 Association between developmental quotient (DQ) and chronological age (months) and the interaction with vitamin breastfeeding indicators for children 1-5 and 1-23 months, evaluated in ENANI-2019 (n = 5,766).

Note: Simple linear regression (DQ ~ chronological age) with breastfeeding indicators interaction: A) No-BF (reference); Exclusive BF: β: -0.076; CI 95%= -0.284-0.132; p=0.473; Predominant BF: β: 0.031; CI 95%= -0.153-0.217; p= 0.738; Mixed BF: β: 0.021; CI 95%= -0.1422-0.184; p= 0.802; Complementary feeding: β: 0.135; CI 95%= -0.042-0.313; p= 0.135. B) No-EBF (reference); EBF: β: -0.141; CI 95%= -0.303 - 0.0196; p= 0.085. C) No-breastmilk (reference); Breastmilk: β: -0.022; CI 95%= -0.034- -0.011; p=0.001. D) Baby bottle use (reference); No- Baby bottle use: β: -0.013; CI 95%= -0.024- -0.001; p=0.027.

The DQ was determined using the milestones questionnaire from the Brazilian adaptation of the Survey of Well-being of Young Children (SWYC-BR). Estimating developmental age considers the child's chronological age and the achieved developmental milestones. The Development Quotient (DQ) is computed by dividing the developmental age by the chronological age. A DQ=1 means the attainment of expected age milestones, while DQ < 1 and > 1 indicate that specific age milestones were reached below or above expectations, respectively. A) The World Health Organization (WHO) BF indicator was defined with the following five categories: a) Exclusive BF – received only breastmilk, without tea, juice, or water; b) Predominant BF – received breastmilk, water, teas and/or juice; c) Mixed BF – received breastmilk and infant formula, regardless of the consumption of water, juice and/or tea; d) Complementary feeding – received breastmilk and any solid or semi-solid food; e) No-BF- does not receive breastmilk. B) The breastmilk intake indicator refers to whether the child received breastmilk or not, regardless of other foods/drinks. C) The baby bottle use refers to who the baby bottle uses on the day before the interview.
